# Supplementary material for: MetaRibo-Seq measures translation in microbiomes
Source: Nat Commun. 2020 Jun 29;11:3268. doi: 10.1038/s41467-020-17081-z (PMC7324362; doi:10.1038/s41467-020-17081-z)
Supplement: Supplementary file 10 — Supplementary Data 7 [file 41467_2020_17081_MOESM10_ESM.zip › File2/Confidence_High_Taxonomy/200103_out.krona.html]

Javascript must be enabled to view this page.

members
magnitude
magnitudeUnassigned
count
unassigned
taxon
rank

200103\_out

80

superkingdom
2
80

phylum
976
80

200643
80
class

order
171549
80

80
815
family

genus

SRS013476\_contig\_number\_8981SRS024140\_contig\_number\_contig-100\_15359.15359SRS056259\_contig\_number\_contig-100\_2502.37531SRS147139\_contig\_number\_24179
816
80
4

species

SRS015890\_contig\_number\_2293SRS017103\_contig\_number\_26629SRS048981\_contig\_number\_contig-100\_1252.51738SRS101433\_contig\_number\_contig-100\_3609.104619SRS1041137\_contig\_number\_contig-100\_5465.154772SRS148159\_contig\_number\_24966SRS893295\_contig\_number\_7398SRS893300\_contig\_number\_contig-100\_3191.73750
671267
8

357276
38
species

SRS011134\_contig\_number\_49550SRS012273\_contig\_number\_16025SRS013098\_contig\_number\_9011SRS013940\_contig\_number\_11822SRS017821\_contig\_number\_27821SRS018049\_contig\_number\_777SRS018541\_contig\_number\_3981SRS018575\_contig\_number\_6719SRS019286\_contig\_number\_5276SRS020869\_contig\_number\_33460SRS022609\_contig\_number\_29658SRS023346\_contig\_number\_643SRS024435\_contig\_number\_15283SRS024549\_contig\_number\_2184SRS045195\_contig\_number\_6299SRS045713\_contig\_number\_11584SRS045826\_contig\_number\_24513SRS052078\_contig\_number\_2770SRS054590\_contig\_number\_69SRS057717\_contig\_number\_5713SRS058070\_contig\_number\_26346SRS058770\_contig\_number\_22495SRS063040\_contig\_number\_483SRS075021\_contig\_number\_781SRS077024\_contig\_number\_535SRS077127\_contig\_number\_1657SRS098655\_contig\_number\_5737SRS098717\_contig\_number\_2966SRS101268\_contig\_number\_500SRS1041112\_contig\_number\_3628SRS104636\_contig\_number\_7634SRS140645\_contig\_number\_341SRS142712\_contig\_number\_1680SRS144135\_contig\_number\_35251SRS146812\_contig\_number\_62136SRS149784\_contig\_number\_19611SRS149879\_contig\_number\_24874SRS150029\_contig\_number\_19340

species

SRS016018\_contig\_number\_1760
1
28116

species

SRS148511\_contig\_number\_12863
1
2292943

1
2292923
species

SRS016381\_contig\_number\_2475

species

SRS018984\_contig\_number\_9097SRS042628\_contig\_number\_34109
457395
2

species

SRS078419\_contig\_number\_8828
1
1263054

species

SRS014613\_contig\_number\_18761SRS048060\_contig\_number\_11019SRS064232\_contig\_number\_2046SRS064276\_contig\_number\_16337SRS075963\_contig\_number\_contig-100\_35.35SRS1041145\_contig\_number\_27429SRS1054691\_contig\_number\_15293SRS1055043\_contig\_number\_11453SRS1055067\_contig\_number\_8943SRS1055099\_contig\_number\_7788SRS147653\_contig\_number\_6645
2292935
11

821
6

SRS013215\_contig\_number\_12SRS024132\_contig\_number\_40335SRS046717\_contig\_number\_7886SRS1055034\_contig\_number\_9215SRS148874\_contig\_number\_4322SRS971276\_contig\_number\_16423
species

6
46506
species

SRS013800\_contig\_number\_19056SRS018133\_contig\_number\_9575SRS024663\_contig\_number\_1628SRS043001\_contig\_number\_3721SRS1041032\_contig\_number\_5576SRS143598\_contig\_number\_7003

species

SRS075341\_contig\_number\_2994
246787
1
